# Diagnostic yield and clinical impact of chromosomal microarray analysis in autism spectrum disorder

**DOI:** 10.1002/mgg3.2182

**Published:** 2023-04-25

**Authors:** Francesca Cucinotta, Carla Lintas, Pasquale Tomaiuolo, Marco Baccarin, Chiara Picinelli, Paola Castronovo, Roberto Sacco, Ignazio Stefano Piras, Laura Turriziani, Arianna Ricciardello, Maria Luisa Scattoni, Antonio M. Persico

**Affiliations:** ^1^ Interdepartmental Program "Autism 0‐90", "G. Martino" University Hospital of Messina Messina Italy; ^2^ IRCCS Centro Neurolesi “Bonino Pulejo” Messina Italy; ^3^ Service for Neurodevelopmental Disorders & Laboratory of Molecular Psychiatry and Neurogenetics University “Campus Bio‐Medico” Rome Italy; ^4^ Mafalda Luce Center for Pervasive Developmental Disorders Milan Italy; ^5^ Synlab Genetics Bioggio Switzerland; ^6^ Neurogenomics Division The Translational Genomics Research Institute Phoenix Arizona USA; ^7^ Research Coordination and Support Service Istituto Superiore di Sanità Rome Italy; ^8^ Child and Adolescent Neuropsychiatry Program, Modena University Hospital & Department of Biomedical, Metabolic and Neural Sciences University of Modena and Reggio Emilia Modena Italy

**Keywords:** 15q11.2–q13.1 duplication syndrome, 16p11.2 microdeletion syndrome, array comparative genomic hybridization, autism spectrum disorder, copy number variants, gene set enrichment analysis, genotype–phenotype correlation, neurodevelopmental disorders

## Abstract

**Background:**

Autism spectrum disorder (ASD) is characterized by high heritability estimates and recurrence rates; its genetic underpinnings are very heterogeneous and include variable combinations of common and rare variants. Array‐comparative genomic hybridization (aCGH) offers significant sensitivity for the identification of copy number variants (CNVs), which can act as susceptibility or causal factors for ASD.

**Methods:**

The aim of this study was to evaluate both diagnostic yield and clinical impact of aCGH in 329 ASD patients of Italian descent.

**Results:**

Pathogenic/likely pathogenic CNVs were identified in 50/329 (15.2%) patients, whereas 89/329 (27.1%) carry variants of uncertain significance. The 10 most enriched gene sets identified by Gene Ontology Enrichment Analysis are primarily involved in neuronal function and synaptic connectivity. In 13/50 (26.0%) patients with pathogenic/likely pathogenic CNVs, the outcome of array‐CGH led to the request of 25 additional medical exams which would not have otherwise been prescribed, mainly including brain MRI, EEG, EKG, and/or cardiac ultrasound. A positive outcome was obtained in 12/25 (48.0%) of these additional tests.

**Conclusions:**

This study confirms the satisfactory diagnostic yield of aCGH, underscoring its potential for better, more in‐depth care of children with autism when genetic results are analyzed also with a focus on patient management.

## Graphical abstract

The diagnostic yield of array‐CGH in ASD is quite satisfactory, with pathogenic/likely pathogenic CNVs identified in 50/329 (15.2%) Italian patients. The ten most enriched gene sets identified by Gene Ontology Enrichment Analysis are primarily involved in neuronal function and synaptic connectivity. Almost half of 50 medical exams prescribed due to aCGH outcome were positive, underscoring its potential for better care of children with autism when genetic results are analysed also with a focus on patient management.

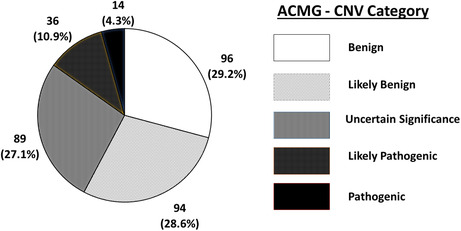

## 1 INTRODUCTION

Autism spectrum disorder (ASD) is a heterogeneous collection of neurodevelopmental conditions with onset in early childhood, characterized by impairment in social interaction and communication, as well as at least two among repetitive behaviors, insistence on sameness, restricted interests, and abnormal sensory processing (American Psychiatric Association, 2013). ASD patients display impressive interindividual differences in clinical symptoms, developmental trajectories, and treatment response (Persico et al., 2020). Despite its high prevalence, no pharmacological treatment effective on core symptoms of ASD has still been found (Persico et al., 2021).

Autism spectrum disorder is considered one of the most “genetic” neuropsychiatric disorders: concordance in monozygotic twins is consistently higher than that observed in dizygotic twins (Huguet et al., 2016). Similarly, family studies show elevated recurrence rates among siblings and first‐degree relatives of affected children, confirming high heritability, which has been estimated at approximately 80% in cohorts from five different countries (Bai et al., 2019). A specific genetic etiology is identifiable in up to 40% of individuals, including known genetic syndromes, mitochondrial disorders, chromosomal deletions or duplications of largely variable sizes, and disruptive mutations detected by exome and genome sequencing (Genovese & Butler, 2020; Schaefer & Mendelsohn, 2013). The majority of cases display complex gene x gene interactions involving multiple common and rare variants, the former endowed with variable penetrance (Bai et al., 2019; Genovese & Butler, 2020; Schaefer & Mendelsohn, 2013). For many patients, also gene–environment interactions involving a genetic predisposition conferred by common variants are plausible (Fernandez & Scherer, 2017). In addition, genetic variants can also contribute to explain interindividual variability in clinical phenotype, developmental trajectories, and responsiveness to behavioral or pharmacological treatment (Cucinotta et al., 2020; Vorstman et al., 2014). Collectively, genetics can thus provide precious information above and beyond “what caused the disorder”, ultimately promoting better care for children with ASD (Butler et al., 2022).

The advent of microarray‐based comparative genomic hybridization (aCGH) technology has unveiled many submicroscopic copy number variations (CNVs) associated with ASD (Devlin & Scherer, 2012). Research studies have shown that clinically relevant CNVs are detected in 9.3–29.0% of patients with idiopathic ASD (Battaglia et al., 2013; Nicholl et al., 2014; Pellanda et al., 2015; Rosenfeld et al., 2010; Tammimies et al., 2015), a substantially higher diagnostic yield compared to conventional karyotyping (Shen et al., 2010). Research data from array‐CGH are important, on the one hand, to define the etiology of autism, since both rare and common CNVs can contribute to cause the disorder, and on the other hand, to outline the functional gene networks involved in the underlying pathophysiology (Gaugler et al., 2014; Grove et al., 2019; Pinto et al., 2014). Therefore, the International Standards for Cytogenomic Arrays (ISCA) Consortium has recommended chromosomal microarray as the first‐tier clinical diagnostic test for children with ASD and various developmental disorders already since 2010 (Miller et al., 2010). However, moving beyond the diagnostic yield, the potential roles of genetic testing by array‐CGH in promoting better clinical management of ASD patients have not yet been directly assessed.

The aim of the present study is twofold: on the one hand, we wish to identify and characterize pathogenetically relevant CNVs in a reasonably sized cohort of Italian ASD patients; on the other hand, we aim to explore whether and to what extent array‐CGH results can contribute to improve the clinical management of autistic patients.

## 2 MATERIALS AND METHODS

### 2.1 Sample

The sample population consisted of 329 idiopathic ASD patients (277 M, 52 F; M:F ratio = 5.3) belonging to 310 families (263 simplex and 47 multiplex). Patients were recruited at the Service for Neurodevelopmental Disorders at Campus Bio‐Medico University Hospital in Rome (Italy) and at the Interdepartmental Program “Autism 0–90” of the “G. Martino” University Hospital (Messina, Italy) between the years 2012 and 2019. All patients fulfilled DSM‐5 criteria for a clinical diagnosis of ASD (American Psychiatric Association, 2013). Developmental, clinical, and family history variables were characterized using an ad hoc questionnaire. Patients with known genetic syndrome or a positive karyotype were excluded. Also patients with major dysmorphisms and malformations were excluded, even in the absence of a genetic diagnosis. Patients with sporadic seizures (<1 every 6 months) were included, whereas epileptic encephalopathy or severe perinatal brain damage documented by MRI were causes for exclusion. The clinical diagnosis of ASD was confirmed in all patients using both the Autism Diagnostic Observation Schedule (ADOS, ADOS‐2) (Lord et al., 2012) and the Autism Diagnostic Interview‐Revised (ADI‐R) (Rutter et al., 2003); cognitive level was assessed using either the Wechsler Intelligence Scales for Children (WISC‐III, WISC‐IV) (Wechsler, 2003), Griffith Mental Developmental Scales II (Huntley, 1996), Colored Raven Matrices (Heinz Wiedl & Carlson, 1976), Leiter International Performance Scale R, or Leiter International Scale—third edition (Roid & Koch, 2017), depending on age and language development. Adaptive behaviors were assessed using the Vineland Adaptive Behavior Scales (Sparrow et al., 1984). All parents gave written informed consent for themselves and for their children. The consent form and all the methods of the study were approved by the Institutional Review Board of University “Campus Bio‐Medico” of Rome, Italy (prot. n. 14/98, first approval on April 28, 1998 and subsequent amendments) and the Ethics Committee of Messina, Italy (prot. n 22/17, approved on June 19, 2017). All methods were carried out in accordance with relevant guidelines and regulations.

### 2.2 Microarray‐based CGH and data analysis

Blood was drawn into EDTA‐anticoagulated tubes from the autistic proband, both parents and unaffected siblings, whenever available. Genomic DNA was extracted and array‐CGH was performed as previously described (Lintas et al., 2017), using the Human Genome CGH SurePrint G3 Microarray 4 × 180 K Kit (Agilent), consisting of ∼170.000 60‐mer oligonucleotide probes which span the whole genome with an average spatial resolution of ∼50 Kb. Following the manufacturer's instructions, 200 ng aliquots of genomic DNA from the test and the sex‐matched reference samples were digested with AluI and RsaI (restriction enzymes). DNA aliquots were then labeled with fluorescent nucleotides (Cy3 and Cy5, respectively) and hybridized for 24 h with an equivalent amount of Cy3‐ and Cy5‐labeled DNA into the microarrays. Slides were finally washed according to manufacturer's instructions and scanned immediately using the DNA Microarray Scanner (Agilent). Quality control was performed using the Agilent Feature Extraction v10.7, and CNV call was performed using the ADM‐2 algorithm, as implemented in the Agilent Cytogenomic Software v.4.0.3.12 and considering aberrations with at least three consecutive probes. All calls were visually inspected to remove possible false positives characterized by irregular Log2 ratios. In order to ensure reliability, CNVs were defined applying the following parameters: minimum number of probes = 3; if 0 = 2 alleles, mean deletions log_2_ ratio < −0.60, and mean duplication log_2_ ratio > +0.54. De novo CNVs and potentially relevant inherited CNVs with ambiguous Log2 ratio profiles were validated by RT‐PCR using TaqMan assays, whenever available, or selective PCR amplification and SybrGreen.

### 2.3 CNVs interpretation

Copy number variations (CNVs) were classified into “rare” or “common” using an R script developed ad hoc, based on the presence of <3 or >3 healthy subjects, respectively, in the last release of the Database of Genomic Variants (DGV) (http://dgv.tcag.ca/dgv/app/home) (MacDonald et al., 2014). Each array‐CGH data output was first blindly classified by four authors (FC, AMP, CL, PT) independently. CNVs were classified in accordance with the American College of Medical Genetics and Genomics (ACMG) and the Clinical Genome Resource (ClinGen) recommendations, as follows: 1 = benign; 2 = uncertain clinical significance; likely benign; 3 = uncertain clinical significance (no subclassification); 4 = uncertain clinical significance—likely pathogenic; 5 = pathogenic (Riggs et al., 2020). Each patient was distributed into one of these five main categories based on the CNV with the highest causative value. Whenever ratings were discordant, investigators discussed the result and reached a consensus. Subsequently, an additional round of analysis was run by another independent rater (MB) using the following software: https://cnvcalc.clinicalgenome.org/cnvcalc/ (Riggs et al., 2020), https://phoenix.bgi.com/autocnv/ (Abou Tayoun et al., 2018) and http://autopvs1.genetics.bgi.com/ (Xiang et al., 2020). Few discrepancies with scores from the first round were detected and further discussed until final consensus was reached. Each patient was ultimately allocated into one of the five categories based on the most pathogenic CNV detected in his/her genome. The following databases were used to collect information about the genes spanned by each CNV: the UCSC Genome Browser (http://genome.ucsc.edu), DECIPHER (https://decipher.sanger.ac.uk/), OMIM (https://www.omim.org), ClinGen (https://www.clinicalgenome.org/), Orphanet (https://www.orpha.net/consor/cgi‐bin/index.php), ISCA (https://isca.genetics. emory.edu), SFARI Gene (https://sfari.org/), AutismKB 2.0 (Yang et al., 2018), GeneCards (http://www.genecards.org/), related literature, and PubMed (https://www.ncbi.nlm.nih.gov). All chromosome coordinates refer to hg19 /GRCh37.

Following genetic testing, patients were clinically reassessed and further medical testing based on the outcome of array‐CGH was prescribed, whenever appropriate.

### 2.4 Gene set enrichment analysis (GSEA) and gene ontology

All genes spanning rare CNVs classified as either “pathogenic”, “likely pathogenic”, or “uncertain clinical significance” were selected, in addition to all genes spanning common CNVs and listed in the SFARI Gene database (“autism genes”). The open‐access web platform Gene Set Enrichment Analysis (GSEA) (http://software.broadinstitute.org/gsea/index.jsp) was then used to perform Enrichment Analysis with the Gene Ontology Functional database (Subramanian et al., 2005), applying a hypergeometric statistics. The FDR method was used to correct for multiple testing, setting statistical significance at FDR <0.05, and then exploring the dataset C5 from the Molecular Signature Database v7.2 (https://www.gsea‐msigdb.org/gsea/msigdb/) to select the top 10 most significant categories. In addition, pathway analysis was performed with R, using specific functions implemented in the Bioconductor package clusterProfiler version 4.6.2 1. The specific function *groupGO*() was used. In this analysis, we considered the more restrictive Gene Ontology levels 4 and 5. Other statistical analyses were performed using the IBM Statistical Package for Social Science (SPSS), version 19.0.

## 3 RESULTS

### 3.1 CNV analysis

**FIGURE 1:**
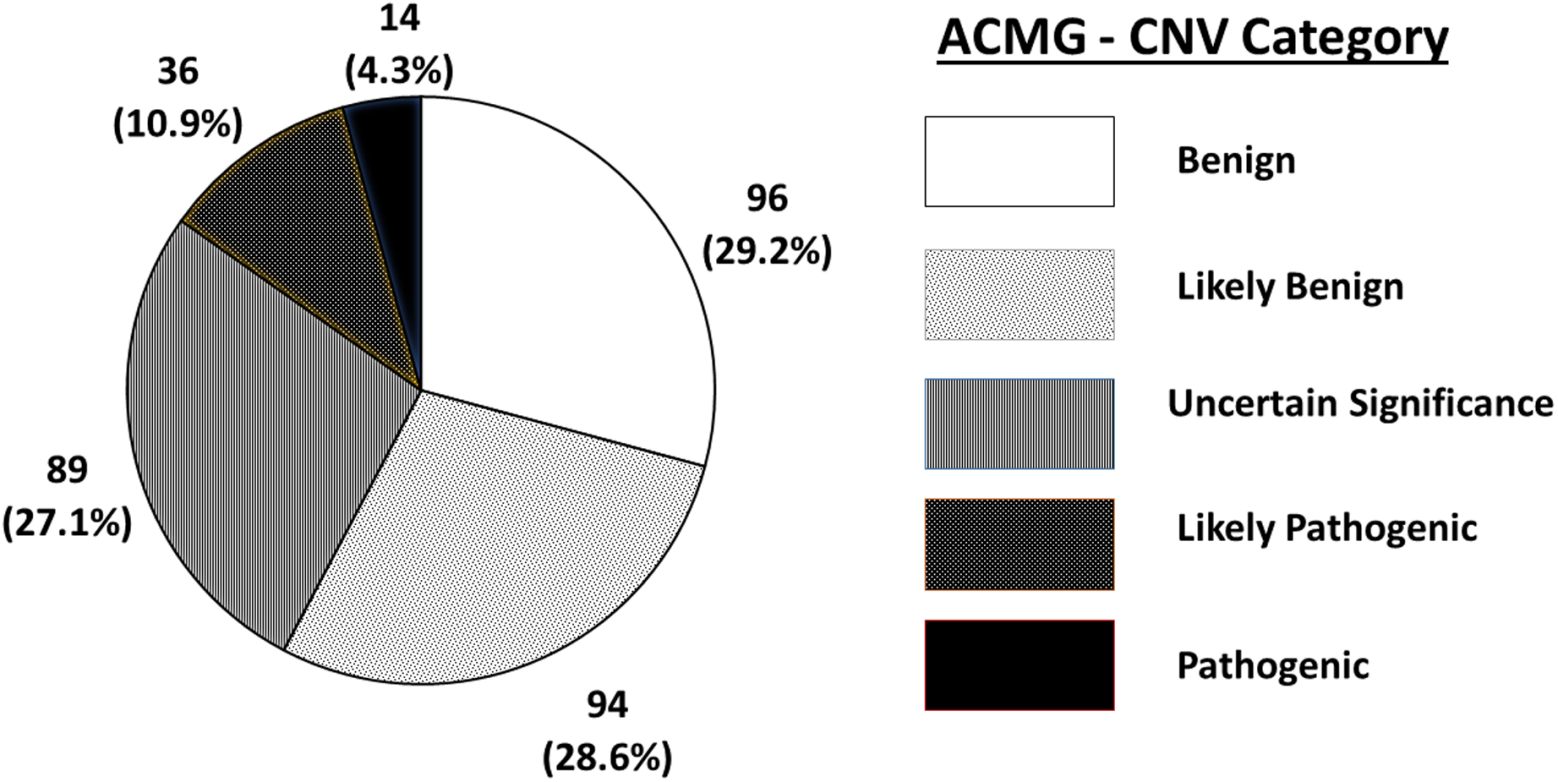
Copy number variants (CNV) classification in accordance with the American College of Medical Genetics and Genomics (ACMGS) and the Clinical Genome Resource (ClinGen) recommendations (Riggs et al., 2020). *N* (%) of patients in each CNV class is specified.

**FIGURE 2:**
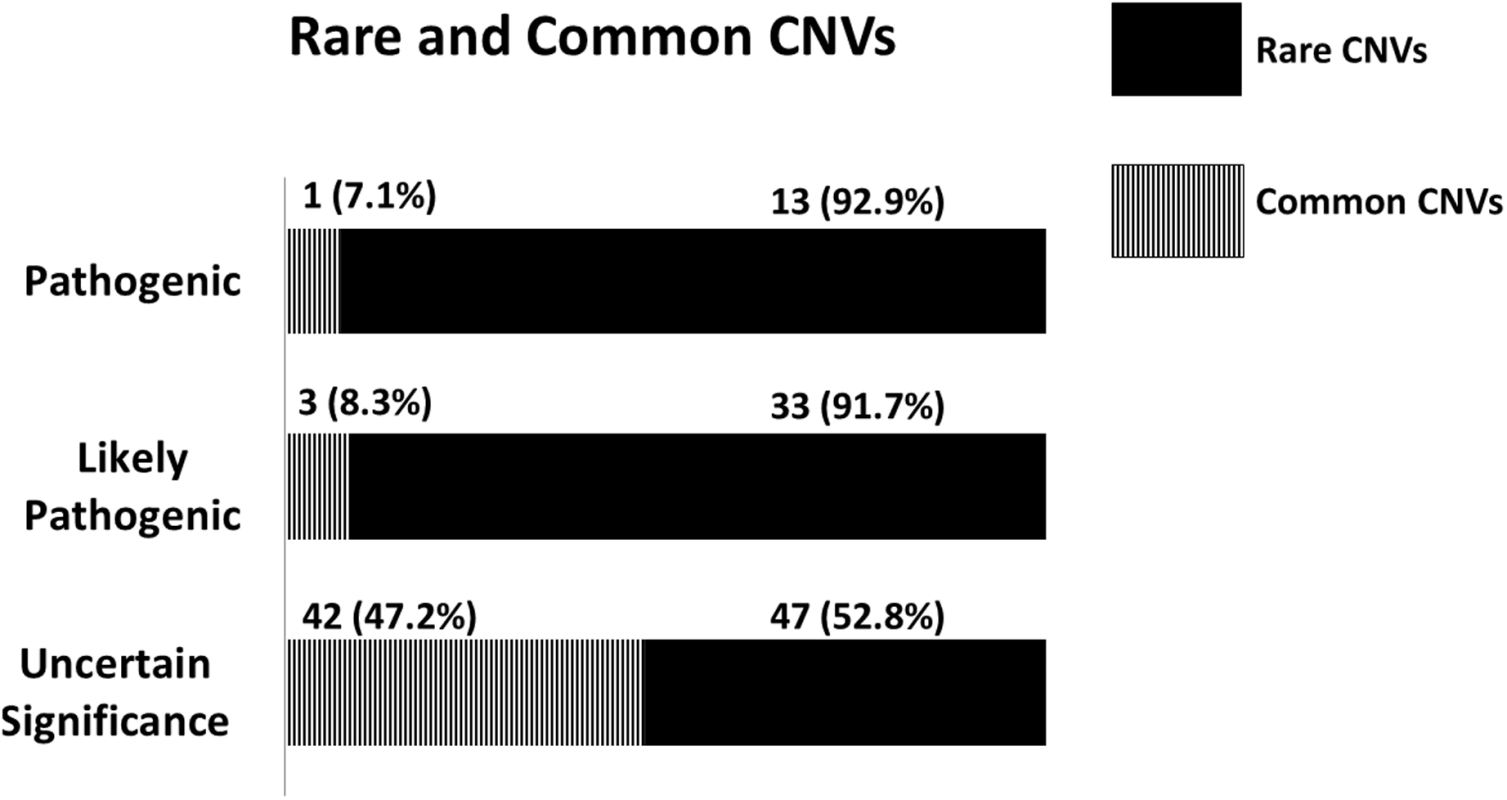
Significantly higher frequency of rare copy number variants (CNVs) in the “pathogenic” and “likely pathogenic” classes, as compared to “variants of uncertain significance”, where rare and common variants are equally distributed (Fisher's exact *p* < 0.00001).

**FIGURE 3:**
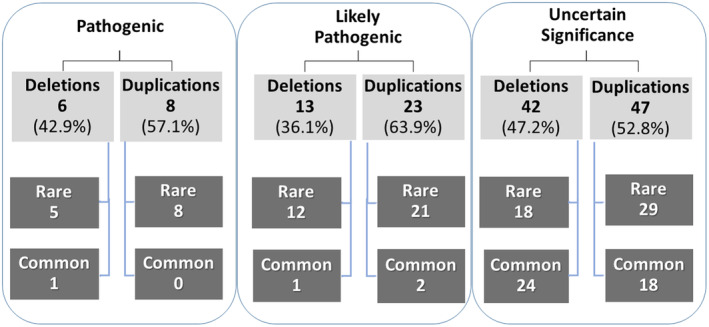
Partitioning of rare and common duplications and deletions among “pathogenic”, “likely pathogenic”, or “uncertain significance” copy number variants (CNVs).

**FIGURE 4:**
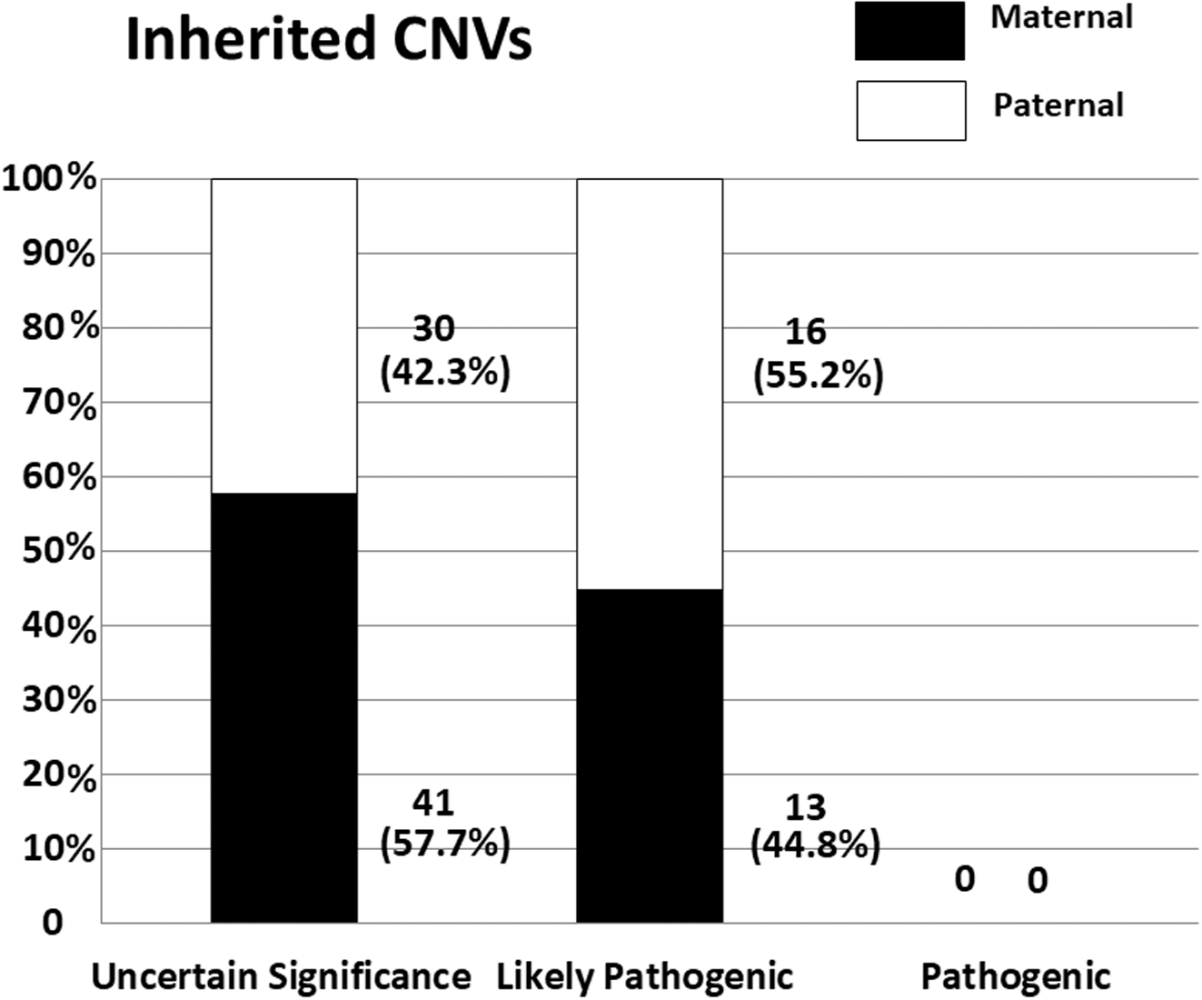
Maternal and paternal inheritance among rare and common “pathogenic”, “likely pathogenic”, or “uncertain significance” copy number variants (CNVs).

The outcome of aCGH analysis is displayed in Figure 1, while a complete list of CNVs with the highest causative value for each one of the 329 autistic individuals enrolled in this study is provided in Table S1. Pathogenic/likely pathogenic CNVs were identified in 50/329 (15.2%) ASD patients (*n* = 14 and 36, respectively). Variants of uncertain significance (VUS) were detected in 89/329 (27.1%) patients. Benign or likely benign CNVs profile was recognized in 190/329 (57.8%) patients (*n* = 94 and 96, respectively). Focusing on the top three categories, the “pathogenic” or “likely pathogenic” classes encompassed, as expected, significantly higher frequencies of rare CNVs compared to the VUS class (46/50 vs. 47/89, Fisher's exact *p* < 0.00001; Figures 2 and 3). Instead, the frequencies of deletions and duplications in the “pathogenic”, “likely pathogenic”, and VUS classes were comparable [χ^2^(2df) = 1.2845, *p* = 0.53, n.s.] (Figure 3), yielding a total of 61/139 (43.9%) deletions and 78/139 (56.1%) duplications. Among these 139 CNVs, 24 (17.3%) were *de novo* and 115 (82.7%) were inherited. “Pathogenic” variants were significantly enriched in *de novo* CNVs, compared to “likely pathogenic” variants and VUS either analyzing rare and common CNVs together [χ^2^(2df) = 24.2203, *p* < 1 × 10^−5^], or analyzing rare deletions and duplications separately (*p* < 0.01 and <0.05, respectively; Table 1a,b). Two patients (n. 436 and n. 218) carry rare de novo deletions located in two regions commonly associated with high susceptibility to neurodevelopmental disorders, namely 16p11.2 (“16p11.2 microdeletion syndrome”, OMIM #611913) (Shinawi et al., 2010) and 17q11.2 (“17q11.2 deletion syndrome”, OMIM #613675) (Osio et al., 2018), respectively. Inherited CNVs were only found among “likely pathogenic” variants and VUS, with no evidence of preferential inheritance from the maternal or paternal side (Figures 4 and S1).

**TABLE 1:** Inheritance patterns among: (a) rare and common CNVs, and (b) rare deletions and duplications, defined “pathogenic”, “likely pathogenic”, or of “uncertain significance” based on ACMG criteria (Riggs et al., 2020).

a) CNVs	De novo	Inherited	NA
Uncertain significance	10/89 (11.2%)	71/89 (79.8%)	8/89 (9.0%)
Likely pathogenic	5/36 (13.9%)	29/36 (80.5%)	2/36 (5.6%)
Pathogenic	9/14 (64.3%)	0/14	5/14 (35.7%)
	χ^2^(2df) = 24.2203, *p* < 1 × 10^−5^		

b) CNVs	De novo	Inherited	NA
Rare deletions			
Uncertain significance	3/29 (10.3%)	24/29 (82.8%)	2/29 (6.9%)
Likely pathogenic	3/21 (14.3%)	16/21 (76.2%)	2/21 (9.5%)
Pathogenic	4/8 (50.0%)	0/8	4/8 (50.0%)
	χ^2^(2df) = 7.1119, *p* = 0.0285		
Rare duplications			
Uncertain significance	2/18 (11.1%)	13/18 (72.2%)	3/18 (16.7%)
Likely pathogenic	2/12 (16.7%)	10/12 (83.3%)	0/12
Pathogenic	4/5 (80.0%)	0/5	1/5 (20.0%)
	χ^2^(2df)=10.9285, *p* = 0.0042		

*Abbreviations*: NA, not available.

Recurrent or common CNVs with an ACMG score equal or higher than 3 are listed in Table 2. Five individuals (patient n. 98, 277, 297, 338, 462) carry a rare “pathogenic” de novo duplication located in chr 15q11.2–q13.1 (“15q11.2‐q13.1 duplication syndrome”, OMIM #608636) (Urraca et al., 2013). Among common CNVs with known or probable functional roles, the 15q11.2 BP1‐BP2 CNV encompassing *TUBGCP5*, *CYFIP1*, *NIPA2*, and *NIPA1* was detected in four patients (n. 118, 177, 222, 235) (Picinelli et al., 2016). Other common duplications and/or deletions, each carried by up to 10 different ASD patients, were found in genes identified as “strong candidates” for ASD (score 2) on the SFARI Gene database, including *CTNNA3* (Wang et al., 2009), *MACROD2* (Anney et al., 2010), *IMMP2L* (Maestrini et al., 2010), *PARK2* (Glessner et al., 2009), *LZTS2* (Wang et al., 2009), and *LRP1* (De Rubeis et al., 2014; Tables 2 and S1). These CNVs are commonly found in the general population and are associated with reduced penetrance (Anney et al., 2010; De Rubeis et al., 2014; Glessner et al., 2009; Maestrini et al., 2010; Wang et al., 2009).

**TABLE 2:** Recurrent copy number variants (CNVs), present in more than one ASD patient.

Chr	Band	Start	End	Size (bp)	Dup/Del	Transmission (n)	Genes (*OMIM n.)	ACMG score
1	p13.3	108,713,464	108,900,263	186,799	Del	pat	SLC25A24 (*608744), NBPF4 (*613994)	3
2	p16.3	51,049,645	51,109,749	60,104	Del	dn (2)	NRXN1 (*600565)	3
3	p26.1	4,013,236	4,290,827	277,591	Del	pat	SUMF1 (*607939)	3
3	q26.31	173,253,431	173,278,401	24,970	Dup	dn (1), pat (1)	NLGN1 (*600568)	3
6	q26	162,669,371	163,082,662	413,291	Dup	mat	PARK2 (*602544)	3
7	q35	146,389,192	146,464,342	75,150	Del	mat	CNTNAP2 (*604569)	4
8	p22	15,952,011	16,021,744	69,733	Del	mat	MSR1 (*153622)	3
8	q21.11	73,596,927	73,635,693	38,766	Dup	mat	KCNB2 (*607738)	3
9	p24.3	288,161	396,907	108,746	Dup	dn	DOCK8 (*611432)	3
10	q21.3	68,056,989	68,201,703	144,714	Del	mat (2)	CTNNA3 (*607667)	3
10	q21.3	68,087,319	68,110,043	22,724	Del	pat	CTNNA3 (*607667)	3
10	q21.3	68,265,513	68,330,749	65,236	Del	mat	CTNNA3 (*607667)	3
10	q21.3	68,292,412	68,319,026	26,614	Del	pat	CTNNA3 (*607667)	3
10	q21.3	68,394,411	68,438,434	44,023	Del	pat	CTNNA3 (*607667)	3
10	q21.3	68,394,411	68,467,599	73,188	Del	mat	CTNNA3 (*607667)	3
10	q21.3	68,427,754	68,558,659	130,905	Del	mat	CTNNA3 (*607667)	3
12	p13.33	1,950,833	1,982,533	31,700	Del	mat	CACNA2D4 (*608171)	3
12	p12.2‐p12.1	21,017,576	21,404,166	386,590	Del	pat	SLCO1B3 (*605495), SLCO1B7 (*619875), SLCO1B1 (*604843)	3
12	q13.3	56,818,735	56,836,254	17,519	Del	mat	TIMELESS (*603887)	3
12	q23.1	99,984,484	100,021,610	37,126	Del	mat	ANKS1B (*607815)	3
13	q12.11	20,090,055	20,270,847	180,792	Dup	mat	TPTE2 (*606791), MPHOSPH8 (*611626), PSPC1 (*612408)	3
15	q11.1‐q11.2	20,481,702	22,834,164	2,352,462	Del	dn	GOLGA6L6, GOLGA8C, BCL8, POTEB (*608912), NF1P1 (*613113), LOC646214, CXADRP2, LOC727924, OR4M2, OR4N4, OR4N3P, GOLGA8D, GOLGA6L1, TUBGCP5 (*608147)	3
15	q11.2	22,765,628	23,085,096	319,468	Dup	mat	TUBGCP5 (*608147), CYFIP1 (*606322), NIPA2 (*608146), NIPA1 (*608145)	3
15	q11.2	22,765,628	23,179,948	414,320	Dup	mat (1), pat (1)	TUBGCP5 (*608147), CYFIP1 (*606322), NIPA2 (*608146), NIPA1 (*608145)	3
15	q11.2	22,765,628	23,208,901	443,273	Del	mat	TUBGCP5 (*608147), CYFIP1 (*606322), NIPA2 (*608146), NIPA1 (*608145), WHAMML1	3
15	q11.2	24,406,896	24,470,140	63,244	Del	mat	PWRN2 (*611217)	3
15	q13.3	32,021,733	32,510,863	489,130	Dup	mat (1), pat (1)	CHRNA7 (*118511)	3
15	q13.3	32,051,233	32,510,863	459,630	Dup	mat	CHRNA7 (*118511)	3
15	q15.3	43,888,927	43,933,733	44,806	Del	mat	CKMT1B (*123290), STRC (*606440), CATSPER2 (*607249)	3
17	q25.3	77,372,621	77,392,578	19,957	Dup	NA	RBFOX3 (*616999)	3
20	p12.1	14,774,913	14,808,986	34,073	Del	NA	MACROD2 (*611567)	3
20	p12.1	14,799,098	14,824,431	25,333	Del	mat (1), pat (1)	MACROD2 (*611567)	3
20	q13.33	62,893,130	62,949,149	56,019	Dup	pat	PCMTD2 (*620077)	4
22	q11.1	17,068,186	17,290,334	222,148	Dup	mat	CCT8L2, psiTPTE22, XKR3 (*611674)	3
22	q11.21	18,894,835	19,010,508	115,673	Dup	mat (1), pat (1), NA (1)	DGCR6 (*601279), PRODH (*606810), DGCR5 (*618040), DGCR9, DGCR10	3
22	q11.21	18,894,835	19,023,883	129,048	Dup	pat	DGCR6 (*601279), PRODH (*606810), DGCR5 (*618040), DGCR9, DGCR10, DGCR2 (*600594)	3
22	q11.21	19,702,774	19,752,605	49,831	Del	dn	SEPT5 (*602724), GP1BB (*138720), TBX1 (*602054)	5
22	q11.22	22,323,105	22,556,733	233,628	Dup	pat	TOP3B (*603582)	3

*Note*: Transmission: dn, de novo; mat, maternal; pat, paternal. Number of transmissions = 1, unless otherwise specified. ACMG scores: 3 = Uncertain clinical significance (no subclassification); 4 = Uncertain clinical significance—likely pathogenic; 5 = Pathogenic (Riggs et al., 2020).

### 3.2 Gene ontology enrichment analysis

Gene ontology enrichment analysis was performed using 436 unique genes, spanning CNVs scored as “pathogenic”, “likely pathogenic”, or of “uncertain clinical significance” in 134 of the 139 ASD cases carrying these variants (Table 3). Five cases carrying a chr. 15q11.2‐q13.1 duplication were excluded from this analysis, because they alone produced a spurious, extreme enrichment in “Nucleolus” (adj‐*p* = 1.33 e^−41^) and “RNA processing” (adj‐*p* = 3.08 e^−34^) gene sets, essentially due to the SNORD gene cluster spanning these five duplications (Table S2). The top 10 most significant gene ontology categories identified by enrichment analysis in the remaining 134 cases encompassed genes involved in neuronal function and synaptic connectivity, such as neuron projection (adj‐*p* = 9.26 e^−8^), synapse (adj‐*p* = 3.4 e^−7^), and cell–cell signaling (adj‐*p* = 4.75 e^−7^). Many of the genes spanned by these CNVs are already associated with ASD and/or neurodevelopmental disorders. In addition, we conducted a complementary analysis of gene ontology with ClusterProfiler using more restrictive levels for each class. The results of the first 30 classes obtained using level 4 and 5 essentially confirm our initial results, also underscoring the importance of calcium‐binding intracellular proteins, as well as proteins involved in DNA/RNA binding and transcriptional regulation (Tables S3‐S8).

**TABLE 3:** Gene set enrichment analysis (GSEA) performed using 436 unique genes spanning CNVs scored as “pathogenic,” “likely pathogenic,” or of “uncertain clinical significance.”

Gene set name	Genes	# genes in overlap (k)	# genes in gene set (K)	# k/K (%)	p‐value	FDR q‐value
GOCC_PLASMA_MEMBRANE_REGION	*NLGN1, CHRNA7, FARP1, GRIN2A, SLC6A1, STX1A, GRID2, PRRT2, HTR5A, CHRNA10, DLG2, SLC6A11, EXOC3, ROBO2, MYO1D, CNTNAP2, TNIK, FZD9, CACNG5, NRXN1, STX19, CLDN3, MYH10, CLDN4, STIM1, APC, MAPK3, DSP, ABCA7, ARL13B, SLCO1B1, SLCO1B3, SH3YL1, PDZK1, DOCK8, EVC, IDE, MFSD10, SLC9A3, AQP7P3, MYOF, ARHGAP45*	42	1233	3.40	1.7 e^−11^	9.26 e^−8^
GOCC_NEURON_PROJECTION	*NLGN1, CHRNA7, FARP1, GRIN2A, SLC6A1, STX1A, GRID2, PRRT2, HTR5A, CHRNA10, DLG2, SLC6A11, EXOC3, ROBO2, MYO1D, CNTNAP2, PRKN, CYFIP1, CDK5R1, NF1, CORO1A, KIF1A, CPEB3, HRH1, DOC2A, TSPOAP1, HTT, FRMPD4, ANKS1B, PCDH15, PIAS3, SMURF1, RACK1, LIMK1, TAOK2, CRMP1, STRC, KCNB2, TRPM1, EXOC6, CCDC141, SNX14, RBM8A, GRK4*	44	1340	3.30	1.78 e^−11^	9.26 e^−8^
GOCC_SYNAPSE	*NLGN1, CHRNA7, FARP1, GRIN2A, SLC6A1, STX1A, GRID2, PRRT2, HTR5A, CHRNA10, DLG2, SLC6A11, EXOC3, TNIK, FZD9, CACNG5, NRXN1, STX19, PRKN, CYFIP1, CDK5R1, NF1, CORO1A, KIF1A, CPEB3, HRH1, DOC2A, TSPOAP1, HTT, FRMPD4, ANKS1B, PCDH15, PIAS3, ZDHHC15, SYN2, PTPRN2, SEPTIN5, ADD1, PJA2, CAMK4, RPS7, CHN2*	42	1305	3.20	9.79 e^−11^	3.4 e^−7^
GOBP_REGULATION_OF_ANATOMICAL_STRUCTURE_MORPHOGENESIS	*NLGN1, CHRNA7, ROBO2, TNIK, CLDN3, MYH10, CLDN4, STIM1, PRKN, CYFIP1, CDK5R1, NF1, CORO1A, KIF1A, SMURF1, RACK1, LIMK1, TAOK2, ZDHHC15, HHEX, RHOG, ALDOA, CDH4, NTN1, RREB1, DOCK1, FLT4, CD160, PRKCA, PIK3CB, TBX1, FOXP1, PDCD6, MARCHF5, GTF2I*	35	976	3.60	2.34 e^−10^	4.75 e^−7^
GOBP_CELL_CELL_SIGNALING	*NLGN1, CHRNA7, FARP1, GRIN2A, SLC6A1, STX1A, GRID2, PRRT2, HTR5A, CHRNA10, DLG2, TNIK, FZD9, CACNG5, NRXN1, STX19, APC, PRKN, CYFIP1, CDK5R1, NF1, CPEB3, HRH1, DOC2A, TSPOAP1, SMURF1, RACK1, SYN2, PTPRN2, SEPTIN5, HHEX, KCTD13, CAPN10, NPS, PLA2G10, VIPR2, MIR142, EIPR1, MCC, SOX11, FOXL2, WWOX, USP34, TBL1XR1, VGLL4, BICC1, BCL7B*	47	1633	2.90	2.87 e^−10^	4.75 e^−7^
GOCC_POSTSYNAPSE	*NLGN1, CHRNA7, FARP1, GRIN2A, SLC6A1, STX1A, GRID2, PRRT2, HTR5A, CHRNA10, DLG2, SLC6A11, TNIK, FZD9, CACNG5, PRKN, CYFIP1, CDK5R1, CPEB3, HTT, FRMPD4, ANKS1B, ZDHHC15, SYN2, ADD1, PJA2, CAMK4*	27	607	4.45	2.96 e^−10^	4.75 e^−7^
GOBP_CYTOSKELETON_ORGANIZATION	*NLGN1, FARP1, MYO1D, TNIK, CLDN3, MYH10, APC, MAPK3, DSP, PRKN, CYFIP1, CDK5R1, NF1, CORO1A, HTT, LIMK1, TAOK2, CRMP1, ADD1, RHOG, ALDOA, KCTD13, CAPN10, NOTCH2, BCL2, MTM1, GPR35, MIR149, ARHGAP6, ARHGAP28, ELN, CTNNA3, WHAMMP3, DIAPH3, EPB41L4A, MRAS, XPO1, CEP70, TUBGCP5, CLIP2, KIF11, GOLGA8DP, GOLGA8CP, NINL*	44	1468	3.00	3.2 e^−10^	4.75 e^−7^
GOBP_COGNITION	*CHRNA7, GRIN2A, SLC6A1, CNTNAP2, FZD9, NRXN1, ABCA7, PRKN, CYFIP1, NF1, CPEB3, HRH1, HTT, PJA2, CAMK4, NPS, NRXN3, DGCR2, CSMD1*	19	306	6.20	6.17 e^−10^	8.03 e^−7^
GOBP_CELL_PART_MORPHOGENESIS	*NLGN1, CHRNA7, FARP1, ROBO2, CNTNAP2, TNIK, NRXN1, PRKN, CYFIP1, CDK5R1, KIF1A, SMURF1, LIMK1, TAOK2, CRMP1, ZDHHC15, RHOG, CDH4, NTN1, RREB1, PLA2G10, NOTCH2, BCL2, MTM1, NRXN3, SLC25A46, LAMA1*	27	658	4.10	1.7 e^−9^	1.91 e^−6^
GOBP_CELL_MORPHOGENESIS	*NLGN1, CHRNA7, FARP1, ROBO2, CNTNAP2, TNIK, NRXN1, CLDN3, MYH10, CLDN4, ARL13B, PRKN, CYFIP1, CDK5R1, CORO1A, KIF1A, SMURF1, LIMK1, TAOK2, CRMP1, STRC, ZDHHC15, RHOG, ALDOA, CDH4, NTN1, RREB1, DOCK1, PLA2G10, NOTCH2, BCL2, NRXN3, SLC25A46, LAMA1*	34	1004	3.40	1.84 e^−9^	1.91 e^−6^

*Note*: The analysis was performed using the MSigDB database v7.5.1, updated January 2022. Gene set names refer to gene ontology cellular components (GOCC) and biological processes (GOBP).

### 3.3 Array‐CGH and clinical management

Following array‐CGH analysis, the 50 patients carrying “pathogenic” or “likely pathogenic” CNVs were reassessed; in 13/50 patients (26.0%), the outcome of the array‐CGH led to prescribe additional medical exams. In some patients, more than one exam was prescribed, yielding a total of 25 prescriptions of medical procedures, exams, or visits (Table 4). The most prescribed exams were EEG (8/25, 32.0%), blood chemistry tests (5/25, 20.0%), brain MRI (4/25, 16.0%), EKG (2/25, 8.0%), and cardiac ultrasound (2/25, 8.0%). Positive outcomes were obtained in 12/25 (48.0%) of these medical exams, requested primarily on the basis of array‐CGH results (Table 4).

**TABLE 4:** Medical diagnostic exams prescribed specifically on the basis of array‐CGH outcome.

ID N.	Clinical diagnosis	CGH	CNV	Medical tests prescribed	Results
10	ASD	Pathogenic	chr22:19702774–19752605	Cardiac sonogram	Mild tricuspid and mitral valve insufficiency
				Abdominal sonogram	Negative
				Neck vessels ultrasound	Tortuous left internal carotid artery
				Blood exams	Negative
				Urinalysis	Abnormal
143	ASD	Likely pathogenic	chr3:93551618–94259056	Blood exams	Negative
245	ASD, DCD, ADHD	Pathogenic	chr2:50194187–50238567 chr2:50340671–50417720	EEG	Frequent sharp waves of very short duration in frontal regions bilaterally
277	ASD	Pathogenic	chr15:22815306–29060493	EEG	Negative, but scarcely modulated during wake and sleep
				EKG	Incomplete right bundle branch block
				Blood exams	Negative
297	ASD	Pathogenic	chr15:22765628–28535051	EKG	Negative
				Blood exams	Altered monocyte counts and bilirubin levels
				EEG	Sporadic abnormalities in the left FCT regions during sleep
316	ASD	Likely pathogenic	chr6:86155395–86925035	EEG	Slight asymmetry and transient sharp theta activity on bilateral central regions during sleep
330	ASD, DCD	Likely pathogenic	chr17:29241181–29490434	EEG	Negative
331	ASD, GDD	Likely pathogenic	chr17:29241181–29490434	Specialized medical consultations	Negative
338	ASD, ID	Pathogenic	chr15:23662509–28535051	EEG	Negative
				Brain MRI	Negative
343	ASD, ID	Likely pathogenic	chr16:89388113–89461642	Blood exams	Negative (only vitamin D3 below normal levels)
				EEG	Negative
436	ASD	Pathogenic	chr16:29652999–30197341	Cardiac sonogram	Negative
				EEG	Sleep spindle asymmetry
				Brain MRI	Slight reduction in volume of the lower face of the right cerebellar hemisphere on the external side with greater salience of the horizontal fissure. Inflammatory thickening of the mucous membrane of the maxillary sinuses, of some ethmoid cells, and of mastoid cells bilaterally
458	ASD	Likely pathogenic	chr3:71166820–71229451	Brain MRI	Herniation in the occipital foramen of both cerebellar tonsils which exceed Mc Rae's plane by about 7 mm
470	ASD	Pathogenic	chr5:108660671–112844710	Brain MRI	Presence of two circumscribed hyperintense lesional foci in the paratrigonal region bilaterally

*Abbreviations*: ADHD, attention deficit/hyperactivity disorder; ASD, autism spectrum disorder; DCD, developmental coordination disorder; EEG, electroencephalogram; EKG, electrocardiogram; GDD, global developmental delay; ID, intellectual disability; MRI, magnetic resonance imaging.

## 4 DISCUSSION

This paper reports the results of array‐CGH analysis conducted on a sample of 329 Italian children with ASD. To ensure the reliability of our CNV scoring method, we adopted a two‐step approach, first classifying blindly CNVs in accordance with the ACMG and the ClinGen recommendations (Riggs et al., 2020), and then reanalyzing these results using publicly available software. Using this approach, we reached a total detection rate of 15.2% “pathogenic” and “likely pathogenic” variants, which is fully comparable with previously reported diagnostic yields ranging between 9.3% and 29% (Battaglia et al., 2013; Nicholl et al., 2014; Pellanda et al., 2015; Rosenfeld et al., 2010; Tammimies et al., 2015). Predictably, rare and *de novo* CNVs are associated with greater pathogenicity, as compared to common and inherited variants, while neither deletions nor duplications are significantly predominant. This may partly stem from the methodological approach, whereby CNVs inherited from an apparently unaffected parent or overlapping with common population variation receive a lower score, according to ACMG recommendation (Riggs et al., 2020). However, we tried as much as possible to determine ACMG scores based on the intrinsic features of the CNV, rather than relying largely on the “de novo” vs. “inherited” criterion, because neurodevelopmental disorders are enriched with inherited pathogenic variants with reduced penetrance and no clear parental expression, as well as with pathogenic epimutations in the proband. At the same time, it is biologically plausible that these variants may be endowed with lower penetrance, while rare and de novo variants, especially those affecting neuronal genes, in different samples typically explain the presence of ASD in ∼5–10% of cases (Autism Genome Project Consortium et al., 2007; Marshall et al., 2008; Pinto et al., 2010). Unfortunately, follow‐up information regarding additional genetic testing performed using NGS is not available, so we do not know how many cases were explained by variants uncovered performing whole exome sequencing.

Among rare variants found in this study, several represent recurrent CNVs in the autism literature or variants of clinical interest. The 15q11.2‐q13.1 duplication syndrome involves several genes implicated in autism, playing key roles in neurodevelopment and specifically expressed in the central nervous system, for example, *ATP10A* (OMIM #605855), *UBE3A* (OMIM #601623), and the *GABRB3* (OMIM #137192), *GABRG3* (OMIM #600233), and *GABRA5* genes (Urraca et al., 2013). Other genes perform basic cellular functions known to be involved in ASD, such as RNA processing (*SNRPN*) and protein degradation (*UBE3A, HERC2*). Clinically, our five patients with the 15q11.2‐q13.1 duplication syndrome all show severe deficits in social communication, mild intellectual disability, and sex ratio M:F = 4:1, in line with clinical descriptions of this syndrome (OMIM #608636) (Urraca et al., 2013). The 16p11.2 microdeletion syndrome (OMIM #611913) (Shinawi et al., 2010) and the 17q11.2 deletion syndrome (OMIM # 613675) (Osio et al., 2018), both confer high susceptibility to ASD, developmental delay, and minor craniofacial dysmorphisms (Osio et al., 2018; Shinawi et al., 2010), all features present in our two patients. Finally, patient n. 376 is a 5‐year‐old boy with severe ASD, verbal language impairment and developmental delay, who inherited from an apparently unaffected parent a 65 kb deletion in chr. 13q32.2, involving the *FARP1* gene. We have recently described this case in detail (Cucinotta et al., 2020), because he did not respond to the same early intensive behavioral intervention which was successful in bringing out of the autism spectrum his older brother, who does not carry this deletion. Although this CNV does not overlap nor appear similar to CNVs identified in other autistic patients, this genetic variation was classified as “likely pathogenic” because *FARP1* hemizygosity may represent a plausible candidate to influence neuroplastic responses to therapeutic environmental stimulation. Farp1 is a synaptic scaffolding protein which regulates synapse function and morphology and promotes actin assembly, dendritic growth, and synaptogenesis (Cheadle & Biederer, 2014).

Common variants collectively have been shown to provide large contributions to ASD susceptibility, with each variant exerting a small effect (Devlin & Scherer, 2012; Gaugler et al., 2014; Huguet et al., 2016). However, some common variants provide more sizable contributions, although their penetrance remains relatively low and clinical expression is variable. The 15q11.2 BP1‐BP2 CNV encompassing *TUBGCP5*, *CYFIP1*, *NIPA2*, and *NIPA1*, presented in a previous report (Picinelli et al., 2016), is a paradigmatic example. In another patient (n.10) were detected as many as 11 CNVs that were not present in parental genomes, suggesting a strong tendency to genomic instability. Many of these CNVs encompass genes included in the best‐known lists of candidate genes for autism, including *CTNNA3* (OMIM #607667) (Wang et al., 2009), *MACROD2* (OMIM #611567) (Anney et al., 2010), *IMMP2L* (OMIM #605977) (Maestrini et al., 2010), *PARK2* (OMIM #600116) (Glessner et al., 2009), *LZTS2* (OMIM #610454) (Wang et al., 2009), and *LRP1* (OMIM #107770) (De Rubeis et al., 2014). In addition, *TBX1* (OMIM #602054) (Paylor et al., 2006), which is located in the center of the region associated with DiGeorge syndrome (OMIM #188400), is partially deleted (six deleted exons out of a total of nine exons) in patient n. 10. The Decipher database lists 16 variations containing the *TBX1* gene and associated with autistic disorder; for SFARI Gene database, *TBX1* is a known “syndromic” gene; moreover, there is also a linkage study (International Molecular Genetic Study of Autism Consortium, 1998) that associates the chr. 22q11.21 region with autism.

Gene Ontology enrichment analysis is aimed at identifying and ranking functionally related groups of genes obtained from high‐throughput experiments. In our study, this analysis fully confirms the importance of neuronal genes, especially structural and functional genes involved in neuronal connectivity (Table 3). This outcome fits well with electrophysiological and functional imaging evidence supporting autism as a mainly “developmental disconnection syndrome” characterized by reduced connectivity among distant brain regions, paired with increased local connectivity (Geschwind & Levitt, 2007). In addition to neuronal gene sets, also “transcriptional regulation”, chromatin structure”, and “immune” genes have been reported in several other genomic and transcriptomic studies (De Rubeis et al., 2014; He et al., 2019; Satterstrom et al., 2020; Voineagu et al., 2011). In our sample, the “nucleolus” and “RNA processing” gene sets yielded the most impressive p‐values, when we analyzed all 139 subjects carrying CNVs with scores 3–5 (i.e., VUS, likely pathogenic, certainly pathogenic; Table S2). If we exclude the five cases carrying the chr. 15q11.2‐q13.1 duplication, these two gene sets disappear from the top 10 list (Table 3). We believe this discrepancy documents that in our sample, the association with transcriptional regulation gene sets was being spuriously boosted by chr 15q duplications, in particular due to the entire *SNORD* gene cluster being consistently duplicated in all five cases (Table S1). However, using more stringent levels of analysis, we find a very complex and mixed set of GO categories, which primarily encompass genes encoding calcium‐binding proteins, as well as DNA‐ or RNA‐binding proteins and transcriptional regulators (Tables S3‐S8). This more stringent analysis confirms that transcriptional regulation and chromatin management are involved in ASD genetics, as documented by GSEAs performed in large exome‐sequencing studies (De Rubeis et al., 2014; Satterstrom et al., 2020). Finally, we do not find “immune” genes spanned by putatively pathogenic CNVs, but indeed there is ample evidence of overexpression of immune genes, especially in ASD brains, according to the vast majority of genome‐wide transcriptomic studies (He et al., 2019; Voineagu et al., 2011). Evidently, this overexpression likely represents one of the convergent functional consequences shared by many different autism‐causing gene variants not directly related to immune function per se, although an additional modulation by common genetic and epigenetic variants located in transcriptional regulatory regions of immune genes is quite plausible.

Another aim of the present work was to verify whether and to what extent genetic testing by CGH‐array can contribute to improve the clinical management of autistic patients. Among the 50 patients carrying pathogenic/likely pathogenic CNVs, 13 (26%) underwent additional medical exams spurred by array‐CGH results (Table 4). These ranged from relatively common exams in the neurodevelopmental disorders clinic, like EEG and EKG, to more specific tests, like cardiac or neck ultrasound. These exams were prescribed only because of a‐CGH results. A positive outcome was obtained in almost half of these diagnostic tests and the more specialized exams almost always yielded positive results. This further step in diagnostic sensitivity arising from array CGH analysis, which goes beyond the mere identification of a plausible etiology and provides information able to improve the clinical management of ASD patients, represents an excellent example of “actionable genomics in clinical practice” (Butler et al., 2022). At this moment, CNV‐based or etiology‐based treatment for ASD is still scarce and this is a major limitation of our current medical management of ASD. This upgrade requires at least two components, a genetic analysis of the results performed also with this clinical aim in mind and a strict collaboration between the clinical/molecular geneticist and the child psychiatrist, who are primarily responsible for the genetic testing and for the clinical management of ASD patients, respectively. In the near future, the complexity of merging genetic and molecular information with structural neurodevelopment, neuropsychological and executive functions, cognitive level, emotional reactivity, social adaptation, and the existential trajectory of an autistic person will represent an increasingly exciting challenge. This perspective may likely require novel teaching and training strategies able to reduce the gap between molecules, neural circuits, and the human mind in order to provide more effective and targeted support to individuals with ASD.

## CONFLICT OF INTEREST STATEMENT

The authors declare no conflict of interest.

## AUTHORS' CONTRIBUTIONS

F.C., C.L., M.L.S., and A.M.P. conceived the study and participated in study design; F.C., L.T., A.R., R.S., and A.M.P. collected patients' history, accomplished the medical work‐up, collected blood samples, and performed psychological testing; M.B., C.P., and P.C., performed genomic DNA extraction and a‐CGH laboratory procedures; P.T., I.S.P., and C.L. analyzed a‐CGH data; C.L., P.T., F.C., A.M.P., and M.B. scored a‐CGH; F.C. wrote the first draft of the manuscript; M.L.S. and A.M.P. revised the manuscript. All authors approved the final manuscript as submitted and agree to be accountable for all aspects of the work.

## Supporting information


Table S1.

Table S2.

Table S3‐S8.

Figure S1.

## Data Availability

The full data set that supports the findings of this study is available from the corresponding author upon reasonable request.
