# Supplementary material for: Diagnostic yield and clinical impact of chromosomal microarray analysis in autism spectrum disorder
Source: Mol Genet Genomic Med. 2023 Apr 25;11(8):e2182. doi: 10.1002/mgg3.2182 (PMC10422062; doi:10.1002/mgg3.2182)
Supplement: Supplementary file 2 — Table S2. [file MGG3-11-e2182-s003.docx]

**SUPPLEMENTARY INFORMATION**

**DIAGNOSTIC YIELD AND CLINICAL IMPACT OF CHROMOSOMAL MICROARRAY ANALYSIS IN AUTISM SPECTRUM DISORDER**

Francesca Cucinotta^1,2^^, Carla Lintas^3^^, Pasquale Tomaiuolo^1^, Marco Baccarin^4,5^, Chiara Picinelli^4^, Paola Castronovo^4^, Roberto Sacco^3^, Ignazio Stefano Piras^3,6^, Laura Turriziani^1^, Arianna Ricciardello^1^, Maria Luisa Scattoni^7^, and Antonio M. Persico^8^*

1. Interdepartmental Program "Autism 0-90", "G. Martino" University Hospital of Messina, Messina, Italy.
2. IRCCS Centro Neurolesi “Bonino Pulejo”, Messina, Italy.
3. Service for Neurodevelopmental Disorders & Laboratory of Molecular Psychiatry and Neurogenetics, University “Campus Bio-Medico”, Rome, Italy.
4. Mafalda Luce Center for Pervasive Developmental Disorders, Milan, Italy.
5. Synlab Genetics, Bioggio, Switzerland.
6. Neurogenomics Division, The Translational Genomics Research Institute, Phoenix, AZ 85004, USA.
7. Research Coordination and Support Service, Istituto Superiore di Sanità, Rome, Italy.
8. Child and Adolescent Neuropsychiatry Program, Modena University Hospital & Department of Biomedical, Metabolic and Neural Sciences, University of Modena and Reggio Emilia, Modena, Italy.

^F.C. and C.L. equally contributed to this manuscript

**Running title:** Clinical impact of array-CGH in autism

**Conflict of Interest:** The authors declare no conflict of interest.

***Correspondence:** Antonio M. Persico, Child & Adolescent Neuropsychiatry, Department of Biomedical, Metabolic and Neural Sciences, University of Modena and Reggio Emilia, Via Giuseppe Campi 287, I-41125 Modena, Italy. Phone number +39-059-2055372; fax +39-059-2055625.

Email: antonio.persico@unimore.it

**Funding information:** Italian Ministry of Health (Grant n. NET-2013-02355263).

**Supplementary Table S1 (see excel file).** List of CNVs with the highest causative value and the relative ACMG score, one per patient, as detected by array-CGH analysis in 329 Italian patients with Autism Spectrum Disorder. OMIM genes are highlighted in bold, followed by their OMIM n.

DGV – Database of Genomic Variants

Similar on Decipher – CNV on Decipher with breakpoints very close those of the patient CNV (i.e., different by no more than +/- 20% of the overall length of the patient CNV)

Identical on Decipher – CNV on Decipher with identical breakpoints

**Supplementary Table S2:** Gene Set Enrichment Analysis (GSEA) for gene ontologies of 517 genes spanning CNVs scored as “pathogenic”, “likely pathogenic”, or of “uncertain clinical significance” detected in 139 of the 329 Italian children with ASD assessed in this study. This analysis includes also five cases carrying a chr. 15q11.2-q13.1 duplication, responsible for boosting here the “Nucleolus” and “RNA processing” gene sets. The remaining gene sets in this analysis largely overlap with those obtained excluding these six cases from the GSEA, as presented in Table 2 of the manuscript.

| **Gene Set Name** | **Genes** | **# Genes in Overlap (k)** | **# Genes in Gene Set (K)** | **k/K** | **p-value** | **FDR q-value** |
| --- | --- | --- | --- | --- | --- | --- |
| NUCLEOLUS | *RPS7,WDR36,BUD23,NOP14,SNORD116-1,SNORD116-2,SNORD116-3,SNORD116-4,SNORD116-5,SNORD116-6,SNORD116-8,SNORD116-10,SNORD116-11,SNORD116-12,SNORD116-13,SNORD116-14, SNORD116-15,SNORD116-16,SNORD116-18,SNORD116-20, SNORD116-21,SNORD116-22,SNORD116-23,SNORD116-24, SNORD1165, SNORD115-2,SNORD116-26,SNORD116-27, SNORD115-3, SNORD115-4,SNORD115-5,SNORD115-6,SNORD115-7,SNORD115-8, SNORD115-9,SNORD115-10,SNORD115-11,SNORD115-13,SNORD115-14, SNORD115-15,SNORD115-16,SNORD115-17,SNORD115-20, SNORD115-21,SNORD115-22,SNORD115-23,SNORD115-25, SNORD115-26, SNORD115-30,SNORD115-31,SNORD115-32, SNORD115-33,SNORD115-34,SNORD115-35,SNORD115-37, SNORD115-38,SNORD115-39, SNORD115-40,SNORD115-41, SNORD115-42,SNORD115-44,SNORD116-29,SNORD115-48, SNORD115-24,SNORD115-27,SNORD115-28, SNORD115-45, SNORD116-30,SNORD115-46,SNORD108, SNORD109A, SNORD115-1, SNORD64,SNORD95,SNORA13,SNORD50B, SNORD116-19, PWAR5,SNORD107,NRXN1,CAMK4,NF1,DOC2A, TAOK2,RREB1,PIK3CB, XPO1,SRP19,TRIM41,MPHOSPH8,FHIT,YPEL3,PSPC1,BAZ1B,INO80E,POLR2E,MACROD2,FOXL2NB,HIRIP3* | 99 | 1421 | 6.96 | 1.28E-45 | 1.33E-41 |
| RNA PROCESSING | *RPS7,WDR36,BUD23,NOP14,SNORD116-1,SNORD116-2,SNORD116-3,SNORD116-4,SNORD116-5,SNORD116-6,SNORD116-8,SNORD116-10,SNORD116-11,SNORD116-12,SNORD116-13,SNORD116-14, SNORD116-15,SNORD116-16,SNORD116-18,SNORD116-20,SNORD116-21,SNORD116-22,SNORD116-23,SNORD116-24,SNORD116-25, SNORD115-2,SNORD116-26,SNORD116-27,SNORD115-3,SNORD115-4,SNORD115-5,SNORD115-6,SNORD115-7,SNORD115-8,SNORD115-9,SNORD115-10,SNORD115-11,SNORD115-13,SNORD115-14, SNORD115-15,SNORD115-16,SNORD115-17,SNORD115-20,SNORD115-21,SNORD115-22,SNORD115-23,SNORD115-25,SNORD115-26, SNORD115-30,SNORD115-31,SNORD115-32,SNORD115-33,SNORD115-34,SNORD115-35,SNORD115-37,SNORD115-38,SNORD115-39, SNORD115-40,SNORD115-41,SNORD115-42,SNORD115-44,SNORD116-29, SNORD115-48,SNORD115-24,SNORD115-27,SNORD115-28, SNORD115-45,SNORD116-30,SNORD115-46,SNORD108, SNORD109A, SNORD115-1,SNORD64,SNORD95,SNORA13, SNORD50B,SNORD116-19,PWAR5,SNORD107,CPEB3,RBM8A,NUP98, SYNCRIP,RIOK1, SUPT4H1,NSUN3,SNRPN,SNRNP48,RBFOX3* | 89 | 1392 | 6.39 | 5.92E-38 | 3.08E-34 |
| PLASMA MEMBRANE REGION | *NRXN1,CHRNA7,SLC6A1,GRIN2A,GABRA5,NLGN1,GRID2,GABRB3,GABRG3,CHRNA10,PRRT2,HTR5A,STX1A,FARP1,DLG2,SLC6A11,FZD9,F2R,CACNG5,TNIK,EXOC3,STX19,CNTNAP2,ROBO2,MYO1D,ABCA7,APC,CLDN3,CLDN4,STIM1,SLCO1B1,SLCO1B3,MYH10,ARL13B,DSP,MAPK3,PDZK1,SLC9A3,MFSD10,AQP7P3,DOCK8,EVC,SH3YL1,IDE,ARHGAP45,MYOF* | 46 | 1233 | 3.73 | 3.05E-11 | 8.71E-8 |
| POSTSYNAPSE | *CAMK4,CPEB3,CHRNA7,SLC6A1,GRIN2A,GABRA5,NLGN1,GRID2,GABRB3,GABRG3,CHRNA10,PRRT2,HTR5A,STX1A,FARP1,DLG2,SLC6A11,FZD9,F2R,CACNG5,TNIK,PRKN,CYFIP1,HTT,CDK5R1,FRMPD4,ANKS1B,PJA2,SYN2,ZDHHC15,ADD1* | 31 | 607 | 5.10 | 3.35E-11 | 8.71E-8 |
| SYNAPSE | *RPS7,NRXN1,CAMK4,NF1,DOC2A,CPEB3,CHRNA7,SLC6A1,GRIN2A,GABRA5,NLGN1,GRID2,GABRB3,GABRG3,CHRNA10,PRRT2,HTR5A,STX1A,FARP1,DLG2,SLC6A11,FZD9,F2R,CACNG5,TNIK,EXOC3,STX19,PRKN,CYFIP1,HTT,CDK5R1,FRMPD4,ANKS1B,PJA2,SYN2,ZDHHC15,ADD1,HRH1,TSPOAP1,PCDH15,KIF1A,CORO1A,PIAS3,PTPRN2,SEPTIN5,GPC1,CHN2* | 47 | 1305 | 3.60 | 5.98E-11 | 1.24E-7 |
| NEURON PROJECTION | *NF1,DOC2A,TAOK2,CPEB3,RBM8A,CHRNA7,SLC6A1,GRIN2A,GABRA5,NLGN1,GRID2,GABRB3,GABRG3,CHRNA10,PRRT2,HTR5A,STX1A,FARP1,DLG2,SLC6A11,EXOC3,CNTNAP2,ROBO2,MYO1D,PRKN,CYFIP1,HTT,CDK5R1,FRMPD4,ANKS1B,HRH1,TSPOAP1,PCDH15,KIF1A,CORO1A,PIAS3,SMURF1,RACK1,KCNB2,TRPM1,LIMK1,CRMP1,STRC,SNX14,EXOC6,CCDC141,GRK4* | 47 | 1340 | 3.50 | 1.43E-10 | 2.49E-7 |
| COGNITION | *NRXN1,CAMK4,NF1,CPEB3,CHRNA7,SLC6A1,GRIN2A,GABRA5,FZD9,CNTNAP2,ABCA7,PRKN,CYFIP1,HTT,PJA2,HRH1,NPS,NRXN3,UBE3A,CSMD1,DGCR2* | 21 | 306 | 6.86 | 2.82E-10 | 4.19E-7 |
| SYNAPTIC SIGNALING | *NRXN1,NF1,DOC2A,CPEB3,CHRNA7,SLC6A1,GRIN2A,GABRA5,NLGN1,GRID2,GABRB3,GABRG3,CHRNA10,PRRT2,HTR5A,STX1A,FARP1,DLG2,F2R,CACNG5,STX19,PRKN,CYFIP1,CDK5R1,SYN2,HRH1,TSPOAP1,PTPRN2,SEPTIN5,NPS,MIR142,KCTD13* | 32 | 741 | 2.31 | 1.08E-9 | 1.34E-6 |
| CELL-CELL SIGNALING | *NRXN1,NF1,DOC2A,CPEB3,CHRNA7,LC6A1,GRIN2A,GABRA5,NLGN1,GRID2,GABRB3,GABRG3,CHRNA10,PRRT2,HTR5A,,TX1A,FARP1,DLG2,FZD9,F2R,CACNG5,TNIK,,TX19,APC,PRKN,CYFIP1,CDK5R1,,YN2,HRH1,T,POAP1,PTPRN2,,EPTIN5,,MURF1,RACK1,NP,MIR142,KCTD13,VIPR2,HHEX,PLA2G10,CAPN10,EIPR1,MCC,OX11,WWOX,U,P34,TBL1XR1,FOXL2,BCL7B,VGLL4,BICC1* | 51 | 1633 | 3.12 | 1.25E-9 | 1.34E-6 |
| INTRINSIC COMPONENT OF PLASMA MEMBRANE | *NRXN1,CHRNA7,SLC6A1,GRIN2A,GABRA5,NLGN1,GRID2,GABRB3,GABRG3,CHRNA10,PRRT2,HTR5A,STX1A,SLC6A11,F2R,CACNG5,CNTNAP2,CLDN3,CLDN4,STIM1,SLCO1B1,SLCO1B3,HRH1,PCDH15,PTPRN2,GPC1,KCNB2,TRPM1,NRXN3,VIPR2,CDH4,ATP10A,FLT4,CD160,NOTCH2,GPR35,STX8,KCNIP3,DPP6,GP1BB,SLCO1B7,SPN,ITGA10,PCDH20,ESYT2,MSR1,BFAR,HJV,ESYT3,FOLH1,TPO,TMEM130,GNRHR2* | 53 | 1737 | 3.05 | 1.29E-9 | 1.34E-6 |

**See excel file for:**

**Table S3:** Enrichment analysis performed using ClusterProfiler. A depth level 4 was applied for Biological Processes. The table shows the top 30 classes.

**Table S4:** Enrichment analysis performed using ClusterProfiler. A depth level 5 was applied for Biological Processes. The table shows the top 30 classes.

**Table S5:** Enrichment analysis performed using ClusterProfiler. A depth level 4 was applied for Molecular Function. The table shows the top 30 classes.

**Table S6:** Enrichment analysis performed using ClusterProfiler. A depth level 5 was applied for Molecular Function. The table shows the top 30 classes.

**Table S7:** Enrichment analysis performed using ClusterProfiler. A depth level 4 was applied for Cellular Component. The table shows the top 30 classes.

**Table S8:** Enrichment analysis performed using ClusterProfiler. A depth level 5 was applied for Cellular Component. The table shows the top 30 classes

**Supplementary Figure S1:** Maternal and paternal inheritance among: (A) rare deletions and (B) rare duplication, defined “Pathogenic”, “Likely Pathogenic” or of “Uncertain Significance” based on ACMG criteria.^32^
