# Supplementary figures and images for: Diagnostic yield and clinical impact of chromosomal microarray analysis in autism spectrum disorder
Source: Mol Genet Genomic Med. 2023 Apr 25;11(8):e2182. doi: 10.1002/mgg3.2182 (PMC10422062; doi:10.1002/mgg3.2182)

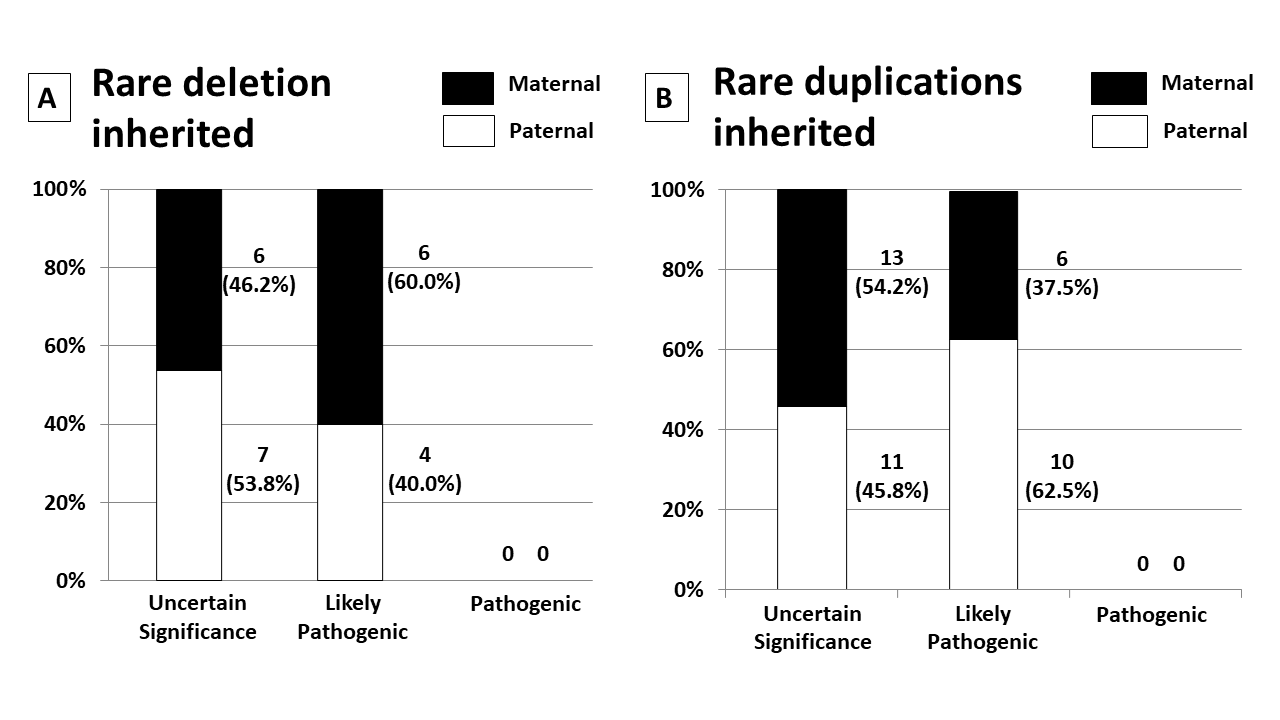

Supplement: Supplementary file 4 — Figure S1. [file MGG3-11-e2182-s004.tif]
